# Cost-effectiveness of brentuximab vedotin in advanced stage Hodgkin’s lymphoma: a probabilistic analysis

**DOI:** 10.1186/s12885-020-07374-3

**Published:** 2020-10-13

**Authors:** A. J. N. Raymakers, S. Costa, D. Cameron, D. A. Regier

**Affiliations:** 1 Health Economics Analytic Support and Research Unit (HEASRU), BC Cancer, Vancouver, Canada; 2 Canadian Centre for Applied Research in Cancer Control (ARCC), BC Cancer Research Centre, 2nd floor, 675 West 10th Avenue, Vancouver, BC V5Z 1L3 Canada; 3 Faculty of Health Sciences, Simon Fraser University, Burnaby, Canada; 4 School of Population and Public Health, University of British Columbia, Vancouver, Canada

**Keywords:** Hodgkin’s lymphoma, Economic evaluation, Brentuximab vedotin, Cost-effectiveness

## Abstract

**Background:**

Treatment with ABVD (doxorubicin, bleomycin, vinblastine, and dacarbazine) is a well-established therapy for advanced Hodgkin’s lymphoma (HL). However, the recently completed ECHELON-1 trial showed potential net clinical benefit for brentuximab vedotin (BREN+AVD) compared to ABVD as frontline therapy in patients with advanced Hodgkin’s lymphoma. The objective of this analysis is to determine whether, on current evidence, BREN+AVD is cost-effective relative to ABVD as frontline therapy in patients with advanced HL.

**Methods:**

We constructed a probabilistic Markov model with two arms and six mutually exclusive health states, using six-month cycle lengths, and a 15-year time horizon. Time-dependent transition probabilities were calculated from ‘real-world’ data collected by the BC Cancer’s Centre for Lymphoid Cancer database or from the literature for ABVD. Time-dependent transition probabilities for BREN+AVD were taken from the ECHELON-1 trial. We estimated the incremental cost and effects per patient of each therapy and calculated the incremental cost-effectiveness ratio (ICER). Costs were measured in 2018 Canadian dollars and effects measured in quality-adjusted life years (QALYs). A probabilistic analysis was used to generate a cost-effectiveness acceptability curve (CEAC).

**Results:**

The incremental cost between standard therapy with ABVD and therapy with BREN+AVD was estimated to be $192,336. The regimen of BREN+AVD resulted in a small benefit in terms of QALYs (0.46 QALYs). The estimated ICER was $418,122 per QALY gained. The probabilistic analysis suggests very few (8%) simulations fall below $100,000 per QALY. Even at a threshold of $200,000 per QALY gained, there was only a 24% chance that BREN+AVD would be considered cost-effective. Sensitivity analyses evaluating price reductions for brentuximab showed that these reductions needed to be in excess of 70% for this regimen to be cost-effective at a threshold of $100,000 per QALY.

**Conclusions:**

There may be a clinical benefit associated with BREN+AVD, but on current evidence the benefit is not adequately substantive compared to ABVD therapy given the cost of brentuximab vedotin. Agencies responsible for making decisions about BREN+AVD as frontline therapy for patients with advanced HL should consider whether they are willing to implement this treatment given the current uncertainty and cost-benefit profile, or negotiate substantial price-reductions from the manufacturer should they choose to reimburse.

## Background

Hodgkin’s lymphoma (HL) is a disease of the lymphatic system, affecting approximately 1000 people per year in Canada [1, 2]. In adults, diagnosis most commonly occurs between ages 20 and 39, or later in life for those over aged 55 years [3].

The prognosis for patients with advanced HL is favorable relative to other advanced cancers. Current evidence suggests that greater than 80% of HL patients will enter a state of complete remission on standard therapy [4, 5]. For patients with advanced HL, the standard therapy is comprised of doxorubicin, bleomycin, vinblastine, and dacarbazine (ABVD). The bleomycin component of ABVD therapy is unpredictable and is often associated with toxicity, and results in adverse events. While the bleomycin component of ABVD therapy has been shown to be associated with toxicity, it should be noted that recent studies have suggested that use of a positron emission tomography (PET) scan to guide treatment might reduce the risk of adverse events, and enable physicians to remove bleomycin from the final cycles of treatment [6]. Also requiring consideration is the fact that while ABVD therapy is well-established, there remains a percentage (30–40%) of patients that will require more aggressive therapies, due to disease relapse or primary refractory disease [7, 8]. Approximately 20% of patients who are not cured with first-line treatment will die after relapse or progression [9].

The ECHELON-1 trial tested brentuximab vedotin plus doxorubicin, vinblastine, and dacarbazine (BREN+AVD) versus ABVD therapy [10]. Brentuximab vedotin is an antibody-drug conjugate that targets the protein CD30 in HL patients [11]. Brentuximab vedotin, in combination with AVD therapy, was examined in the ECHELON-1 trial as a potentially promising treatment that could replace ABVD as standard frontline care for advanced-stage HL patients [10]. The results of the trial showed improved differences for patients treated with BREN+AVD in terms of modified progression free survival (HR: 0.77 (95% CI: 0.60–0.98)) but no statistically significant improvement in overall survival (HR: 0.72 (95% CI: 0.44–1.17)) [10].

While the results of the ECHELON-1 trial suggest a potential net clinical benefit in terms of modified progression free survival from treatment with BREN+AVD, the price of BREN+AVD is substantially higher than ABVD therapy. While there may be evidence of a net clinical benefit associated with BREN+AVD as frontline therapy in patients diagnosed with advanced HL, there remains uncertainty as to whether or not this therapy is cost-effective, particularly from a Canadian perspective, when compared to more established therapies. This analysis reports on the results of a cost-effectiveness analysis of ABVD compared to BREN+AVD used as frontline therapy for patients with advanced stage Hodgkin's lymphoma from the perspective of the Canadian province of British Columbia.

## Methods

### Modeling overview

**Fig. 1:**
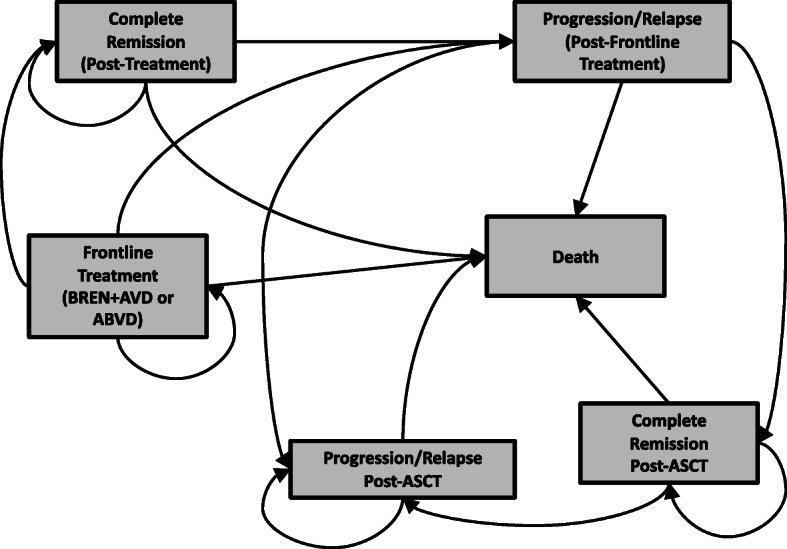
Conceptual diagram of possible model transitions

A state-transition Markov model was constructed with two treatment arms. In one arm, patients would receive therapy with BREN+AVD; in the other, patients would receive the current standard of care, ABVD. Both arms were structurally identical and consisted of six potential health states: (i) treatment with the relevant therapy; (ii) complete remission after initial treatment; (iii) progression or relapse after initial treatment (and potential receipt of autologous stem cell transplant (ASCT)); (iv) remission after second treatment with ASCT; (v) second progression or relapse (resulting in ‘salvage’ therapy with pembrolizumab); and, (vi) death (see Fig. 1). Each of the health states are mutually exclusive – meaning that a patient can only be in one specific health state during each model cycle. Each model cycle was assumed to be 6 months long, and the model time horizon was 15 years. While the structure of the model was identical for both arms, the data used to populate the models (i.e., probabilities, costs) were specific to each of the respective therapies. Additional details of the model structure are available in the Supplementary Material.

Within each specific health state, further events were possible. For example, in the initial treatment cycle, patients were able to experience complete remission, an adverse event, treatment discontinuation, or death. The probabilities of each of these potential events were based on data for that particular therapy and are presented, with sources, in Table 1. The model was used to estimate the costs and effects of the experimental arm (BREN+AVD) and the standard of care arm (ABVD). The incremental costs and incremental effects from each arm were then used to estimate the incremental cost-effectiveness ratio (ICER). The modeling approach taken was consistent with guidelines produced in Canada for the economic evaluation of health technologies by the Canadian Agency for Drugs and Technologies in Health (CADTH) [18]. The model was programmed in TreeAge Pro 2018 (TreeAge Software; Williamstown, USA). Ethics approval for this study was obtained by the University of British Columbia – BC Cancer Research Ethics Board (H18–00490).

**Table 1:** Model parameters, sources, and distributions used in the probabilistic analysis

Parameter	Value	Distribution	Source
**Probabilities**			
Serious adverse event (BREN+AVD)	0.429	Beta	Connors et al. (2018) [10]
Serious adverse event (ABVD)	0.270	Beta	Connors et al. (2018) [10]
Treatment discontinuation (BREN+AVD)	0.133	Beta	Connors et al. (2018) [10]
Treatment discontinuation (ABVD)	0.159	Beta	Connors et al. (2018) [10]
Mortality on treatment (BREN+AVD)	0.013	Beta	Connors et al. (2018) [10]
Mortality on treatment (ABVD)	0.019	Beta	Connors et al. (2018) [10]
Progression/relapse while in complete remission (BREN+AVD)	Time Dep	Beta	Connors et al. (2018) [10]
Mortality in complete remission (BREN+AVD)	Time Dep	Beta	Connors et al. (2018) [10]
Progression/relapse while in complete remission (ABVD)	Time Dep	Beta	CLC
Mortality in complete remission (ABVD)	Time Dep	Beta	CLC
Eligible for ASCT (BREN+AVD or ABVD)	0.8	Beta	Expert Opinion
Mortality in progression/relapse (ABVD)	Time Dep	Beta	Vivani et al. (2011) [12]
Mortality in progression/relapse (BREN+AVD)	Time Dep	Beta	Vivani et al. (2011) [12]
Successful ASCT	0.5	Beta	Expert Opinion
Progression/relapse post-ASCT (BREN+AVD)	Time Dep	Beta	CLC
Progression/relapse post-ASCT (ABVD)	Time Dep	Beta	CLC
Mortality post-ASCT progression/relapse (BREN+AVD or ABVD)	Time Dep	Beta	Chen et al. (2016) [13]
**Costs**			
Cost BREN+AVD	$116,160	Gamma	Canadian list price for six cycles
Cost ABVD	$12,701	Gamma	Canadian list price for six cycles
PET scan	$1877	Gamma	Cerci et al. (2010) [14]
Cost of adverse event (ABVD or BREN+AVD)	$12,036	Gamma	Wong et al. (2018) [15]
ASCT	$67,723	Gamma	Bloomstein et al. (2012) [16]
Salvage chemotherapy for non-responders	$140,800	Gamma	Canadian list price for six cycles
**Utility values**			
Receiving treatment (ABVD or BREN+AVD)	0.71	Beta	Swinburn et al. (2015) [17]
Complete remission	0.91	Beta	Swinburn et al. (2015) [17]
Adverse event	0.59	Beta	Swinburn et al. (2015) [17]
Progressive disease	0.38	Beta	Swinburn et al. (2015) [17]
**Model details**			
Cycle length	6 months		Assumed
Time horizon	15 years		Assumed
Cost year	2018		Assumed
Discount rate: costs	1.5%		Assumed
Discount rate: effects	1.5%		Assumed

*Time Dep* time-dependent transition probability. *CLC* Centre for Lymphoid Cancer

### Transition probabilities

Real-world data to inform the standard of care (ABVD) arm of the model were obtained from the BC Cancer’s Centre for Lymphoid Cancer (CLC) database. The CLC database houses data on treatment, response to treatment, and dates of significant clinical events for patients with lymphoid cancer in the province of BC. Included patients were greater or equal to 18 years of age and were diagnosed between 2000 and 2016 in BC. To avoid potential interactions with other conditions, patients who were HIV-positive and/or pregnant at time of diagnosis were also excluded from our sample. After these criteria were applied, a cohort of 1519 patients was established which was used to calculate transition probabilities for the standard care arm of the model. To derive transition probabilities for standard care (ABVD), we calculated the time elapsed between events of interest reported in the CLC database (i.e., the transitions between health states in the Markov model), and then fit a Weibull distribution to the data. From the fitted Weibull distribution, we then computed probabilistic beta-distributed transition probabilities. For BREN+AVD, time-dependent probabilities were extracted from published data. Details of specific sources for the data are available in Table 1.

To derive transition probabilities for the BREN+AVD arm, the statistical analysis software ‘R’ (Version 3.6.1; Vienna, Austria) and package FlexSurv [19] were used to perform Weibull regression on digitized published data from the ECHELON-1 trial [10]. Variance was calculated using the Hessian at the maximum, transformed back to the original scale of the parameters at each time point [19]. Future transition probabilities were calculated using the calculated Weibull curve and the standard error of prediction at each time point [20, 21]. Additional probabilities were obtained from relevant literature [12, 13] (see Table 1).

### Costs and utilities

The costs for both BREN+AVD and ABVD therapy are based on the Canadian list prices for these regimens. All other costs were taken from the literature [14–16]. The ECHELON-1 trial did not report on costs collected alongside the trial. Costs are presented in 2018 Canadian dollars from the health care payer perspective.

Utility estimates were taken exclusively from the literature, based largely on a study by Swinburn et al. [17]. This study focused on patients specifically with relapsed/refractory disease but provided utility estimates, using the time trade-off (TTO) method, for patients with a complete response, stable disease, experiencing adverse events, and with progressive disease. The study presented data for several countries; in the reference case analysis we have used estimates from the United Kingdom (*n* = 100). All costs and outcomes were discounted at 1.5% per year.

### Model assumptions

Utility values for all health states for ABVD and BREN+AVD are the same. This assumption is largely a result of a dearth of original health-related quality of life (HRQoL) data being collected or reported for HL patients, including HRQoL data being reported from ECHELON-1 [22]. We have assumed a price of BREN+AVD therapy of $116,160 CAD which is based on the list price for six cycles of BREN+AVD therapy. The assumed cost and number of cycles for BREN+AVD is based on the ECHELON-1 trial but this may be variable in practice based on the patient’s age, response to treatment, and toxicity [10, 23]. Recent studies have also suggested the addition of concurrent medications (i.e.*,* pegfilgrastim) to reduce the risk of adverse events, which may double the cost of the therapy [24]. Finally, evidence also suggests that bleomycin may be removed from the final cycles of ABVD therapy which reduces the probability of adverse events, but we have kept bleomycin included in ABVD therapy as a conservative assumption.

### Probabilistic analyses

A probabilistic analysis was conducted using Monte Carlo simulation with 10,000 iterations from appropriate distributions of the input parameters (see Table 1). The probabilistic analysis generated a range of ICERs, which were plotted on the cost-effectiveness plane. The cost-effectiveness plane plots the incremental costs (y-axis) and incremental benefit in terms of QALYs (x-axis) for each model simulation. The probabilistic analysis was also used to generate a cost-effectiveness acceptability curve (CEAC). The CEAC shows the probability of whether or not a particular treatment regimen will be cost-effective at different levels of willingness-to-pay for an additional QALY. Finally, given the considerable variability in reported costs of brentuximab vedotin and the potential for price negotiations if chosen for reimbursement, we conducted specific scenario analyses using different prices of this treatment regimen.

## Results

The results of this economic evaluation suggest a substantial estimated incremental cost between the standard therapy with ABVD and therapy with BREN+AVD ($192,336) and a small benefit in terms of QALYs (0.46 QALYs). This resulted in an estimated ICER of $418,122 per QALY gained (Table 2). While Canada has no explicit willingness-to-pay threshold per QALY gained, this exceeds commonly cited thresholds of $50,000 or $100,000 per QALY gained, indicating that BREN+AVD therapy is unlikely to be cost-effective compared to ABVD therapy.

**Table 2:** Results from the reference case probabilistic analysis (95% confidence intervals)

Treatment	Cost (CAD$)	Effect (QALYs)	Incremental Cost	Incremental Effect	ICER
**BREN + AVD**	$411,190	9.62	$192,336	0.46	$418,122
	($300,490–$554,715)	(7.29–11.0)			
**ABVD**	$218,854	9.16			
	($156,367–$310,743)	(6.98–10.49)			

**Fig. 2:**
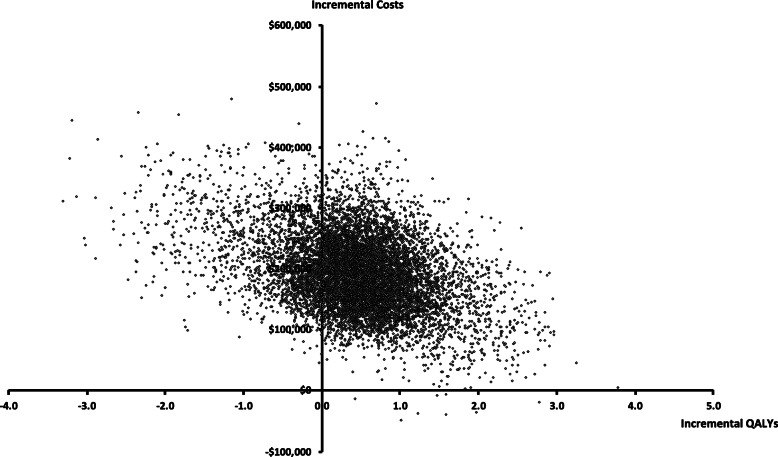
Scatter plot of incremental cost-effectiveness ratios (ICERs) generated from the probabilistic analysis (*n* = 10,000 iterations)

**Fig. 3:**
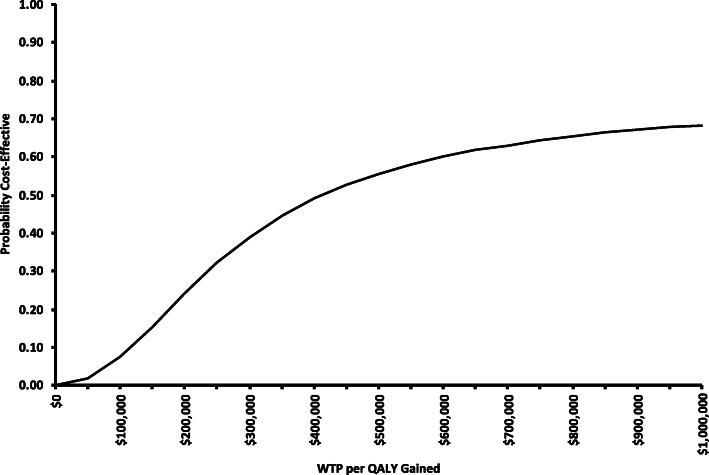
Cost-effectiveness acceptability curve showing the probability that therapy including brentuximab vedotin is cost-effective at various levels of willingness-to-pay (WTP) per QALY gained

The cost-effectiveness plane of each ICER calculation is presented in Fig. 2 and the CEAC is in Fig. 3. The probabilistic analysis showed that only 8% of model simulations resulted in an ICER less than a $100,000 per QALY threshold, and less than 1% simulations were associated with greater effects and lower costs for BREN+AVD versus the standard of care. Importantly, the uncertainty associated with BREN+AVD therapy was demonstrated in that approximately 20% of simulations suggested that this therapy resulted in lower incremental effects and greater incremental costs than ABVD therapy. In Fig. 3, the CEAC shows that when a threshold of $100,000 per QALY gained is used, there is an approximately 10% chance of treatment with BREN+AVD being cost-effective.

Given a potential clinical benefit from BREN+AVD therapy but that our analysis suggested that BREN+AVD was not cost-effective, the price of this therapy was adjusted to evaluate at what cost the therapy could be considered cost-effective relative to ABVD. In this analysis, a greater than 70% reduction in the cost of BREN+AVD was required for this therapy to be a cost-effective alternative to ABVD at a threshold of $100,000 per QALY.

## Discussion

This analysis evaluated the cost-effectiveness of a treatment regimen including brentuximab vedotin, compared to standard therapy with ABVD, as frontline therapy for patients with advanced HL in British Columbia, Canada. The recent phase III trial ECHELON-1 reported that treatment for patients with advanced HL with brentuximab vedotin was shown to offer a net clinical benefit using a surrogate outcome measure (modified progression-free survival) compared to the standard of care (ABVD). However, our economic evaluation has shown that the benefit associated with BREN+AVD therapy is unlikely to be commensurate with the high cost of this therapy. This result is reinforced given that there is reliable long-term real-world evidence for the effectiveness of ABVD therapy, while the evidence for BREN+AVD therapy relies on limited and short-term efficacy data from a phase III trial [10].

Previous economic evaluations of brentuximab vedotin as frontline treatment for advanced HL have come to conflicting conclusions. Delea et al. [25], also using data from the ECHELON-1 trial reported an ICER of $172,074 per QALY (using the overall study population from ECHELON-1) or $69,442 per QALY gained when the study population was restricted to data from North American sites. This analysis assumed favorable benefits for brentuximab vedotin as it relied on key assumptions including investigator-reported data (as opposed to that assessed by an independent review committee) and used modified progression free survival as the effectiveness measure for a select population in North America. Based on these results, the authors concluded that it was likely to be cost-effective in this patient population [25]. In contrast, Huntington et al. [26] conducted a cost-utility analysis based on the results of the ECHELON-1 trial and drew comparable conclusions to our own, finding an ICER of $317,254 per QALY gained, largely due to the small incremental gain in QALYs between BREN+AVD and ABVD (0.56 QALYs). The authors also noted that there was a lack of HRQoL (utility) data for BREN+AVD and ABVD therapy to inform the QALY calculation.

Given the reported net clinical benefit from BREN+AVD therapy [10], an analysis was conducted to determine at what cost BREN+AVD would be cost-effective for the treatment of advanced HL patients. Similar to the analysis conducted by Huntington et al. [26] we carried out a series of sensitivity analyses to determine at what price BREN+AVD is required to be in order to be considered cost-effective at an assumed threshold of $100,000 per QALY gained. It should be noted that other studies have reported higher cost of therapy with BREN+AVD [24]. The implication of a higher cost for BREN+AVD is obvious; the result would be an even lower probability that it would be cost-effective as frontline therapy given the current state of evidence. The value of any new therapy is relative to what is currently used in practice, so the increment that the health care provider should be willing to pay, based on the value, could potentially set the price. Our probabilistic analysis produced an important result. The uncertainty associated with the treatment effect of BREN+AVD resulted in a considerable number of simulations with a negative incremental benefit, favouring treatment with ABVD.

Brentuximab vedotin and the ECHELON-1 trial provide a perfect example of some of the deficiencies of current clinical trials in informing reimbursement decision-making. Frontline therapy for patients with advanced HL, as stated, is well-established. As such, we were able to obtain real-world data from patients treated at BC Cancer to populate our economic model. This can be contrasted with the estimated beneficial effect in ECHELON-1, a small, albeit statistically significant increase in a surrogate outcome measure, modified progression-free survival. The implication is that the use of surrogate outcome measures should be interpreted cautiously, and should not be sufficient to alter clinical practice in place of more established outcomes (i.e., overall survival), which is a better metric of a treatment value [24]. Moreover, if this use of surrogate outcome measures is coupled with a failure to collect (or report) HRQoL data the trial has almost certainly failed to collect data that matters to patients, and by extension, decision-makers.

### Limitations

There are limitations to our study that require acknowledgement. First, several model parameters are based on necessary assumptions for our model. Utility values, for example, used to calculate QALYs were not original data collected alongside the ECHELON-1 trial and were taken from a previous study [17]. To the best of our knowledge, the ECHELON-1 trial did not report on HRQoL data (if collected) that could be used for economic analysis, which is common for oncology clinical trials [27]. Similarly, our analysis assumed that treatment costs were based on Canadian list prices and did not include additional associated costs. Second, economic models representing disease pathways are always a simplification of reality, and there are different trajectories that individual patients might take that are not represented by our model structure. In order to be able to provide economic evidence to decision-makers, however, these simplifications are necessary. We have attempted to mitigate these simplifications by engaging clinical experts in HL to provide face validity to our model. Third, for the time horizon to be suitably long enough given this disease area, we extrapolated reported survival curves to 15 years, but acknowledge that, if the data were available, a lifetime time horizon would be superior. Finally, although we have reported that BREN+AVD is unlikely to be a cost-effective use of health resources, as with any economic evaluation, there are factors beyond what might be included in the analysis, which should be considered in relation to whether or not a drug is ultimately reimbursed.

## Conclusions

For patients with advanced Hodgkin’s Lymphoma, the minimal treatment gains that can be achieved through a therapy containing brentuximab vedotin do not appear to warrant the cost based on current evidence. While ECHELON-1 showed a net clinical benefit in terms of a surrogate endpoint resulting from a therapy with brentuximab vedotin, we have shown that at its current price, it would be an inefficient use of health care resources. Importantly, the manufacturers might heed the results of this study to decrease the price of therapy commensurate to its benefits and continue to collect evidence for its effectiveness. Coupled with high cost of brentuximab is the uncertainty in its use compared to the real-world data of a long established and beneficial therapy (ABVD). This analysis highlights the value of real-world data in helping inform decisions about the funding of new therapies with short-term or limited evidence compared to established therapies.

## Supplementary information

**Additional file 1: Supplementary Figure 1.** Detailed Model schematic. **Supplementary Figure 2.** Overall survival curves extrapolated from the ECHELON-1 trial (Weibull distribution). **Supplementary Figure 3.** Modified progression-free survival curves extrapolated from the ECHELON-1 trial (Weibull distribution).

## Data Availability

The dataset informing the standard of care arm generated and/or analyzed for this study are not publicly available due to privacy restrictions. The remainder of the data is presented in Table 1 of this manuscript or available in the literature.

## Abbreviations

ABVD: Doxorubicin, bleomycin, vinblastine, and dacarbazine
ASCT: Autologous stem cell transplant
BC: British Columbia
BREN+AVD: Brentixmab vedotin, doxorubicin, vinblastine, and dacarbazine
CADTH: Canadian Agency for Drugs and Technologies in Health
CEAC: Cost-effectiveness acceptability curve
CI: Confidence interval
CLC: Centre for Lymphoid Cancer
HL: Hodgkin’s lymphoma
HRQoL: Health-related quality of life
ICER: Incremental cost-effectiveness ratio
QALY: Quality-adjusted life-year
TTO: Time-trade off
