# Supplementary material for: Cost-effectiveness of brentuximab vedotin in advanced stage Hodgkin’s lymphoma: a probabilistic analysis
Source: BMC Cancer. 2020 Oct 13;20:992. doi: 10.1186/s12885-020-07374-3 (PMC7557030; doi:10.1186/s12885-020-07374-3)
Supplement: Supplementary file 1 — Additional file 1: Supplementary Figure 1. Detailed Model schematic. Supplementary Figure 2. Overall survival curves extrapolated from the ECHELON-1 trial (Weibull distribution). Supplementary Figure 3. Modified progression-free survival curves extrapolated from the ECHELON-1 trial (Weibull distribution). [file 12885_2020_7374_MOESM1_ESM.docx]

**Supplementary Material**

**Supplementary Figure 1.** Detailed Model schematic.

The figure below represents the model structure for one arm of the model (the treatment regimen including brentuximab vedotin). The structure of the model is identical for both arms, with the only differences being the variable names and assigned data. The six potential health states are those nodes following the Markov symbol. Within each of these health states there are a variety of subsequent events that can occur within one model cycle (6 months). For example, within the initial treatment with brentuximab, patients may follow four possible paths, including responding to therapy, experiencing adverse events, treatment discontinuation, or death.

**
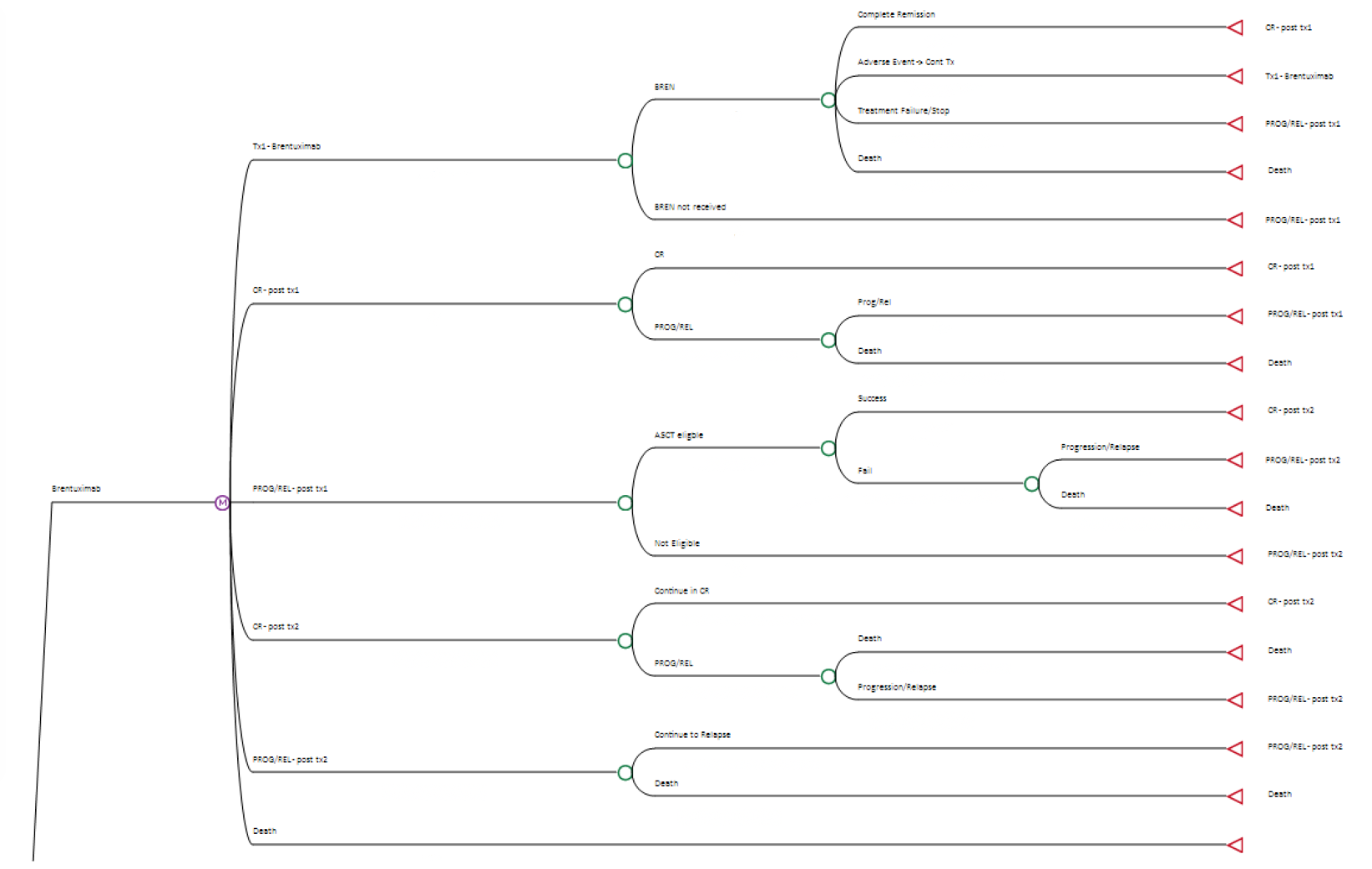
**

**Supplementary Figure 2.** Overall survival curves extrapolated from the ECHELON-1 trial (Weibull distribution).

The red line represents the trial data and the black line the extrapolated data for a period of 180 months.

(a) Overall survival for BREN+AVD therapy based on ECHELON-1.


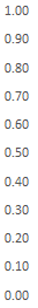


Overall Survival

(b) Overall survival for ABVD therapy based on ECHELON-1.

Overall Survival

**Supplementary Figure 3.** Modified progression-free survival curves extrapolated from the ECHELON-1 trial (Weibull distribution).

The red line represents the trial data and the black line the extrapolated data for a period of 180 months.

(a) Modified progression-free survival for BREN+AVD therapy based on ECHELON-1.

Modified progression-free survival


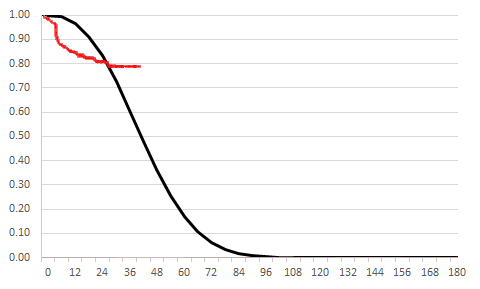


(b) Modified progression-free survival for ABVD therapy based on ECHELON-1.

Modified progression-free survival


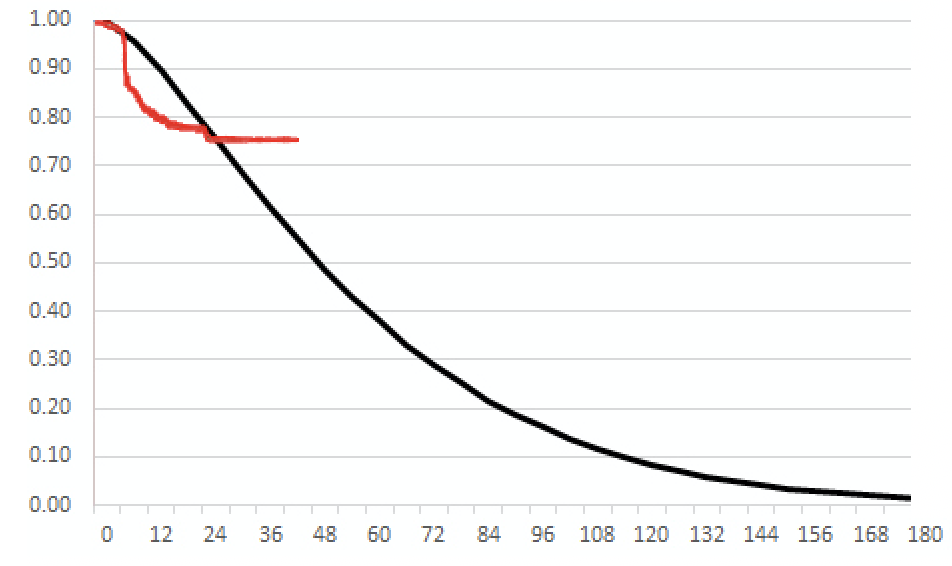


**References:**

Connors JM, Jurczak W, Straus DJ, Ansell SM, Kim WS, Gallamini A, et al. Brentuximab Vedotin with Chemotherapy for Stage III or IV Hodgkin’s Lymphoma. New England Journal of Medicine. 2018 Jan 25;378(4):331–44.
